# Factors Related to Childbirth Intention of Migrant Women in Multicultural Marriages in Korea

**DOI:** 10.18502/ijph.v49i8.3888

**Published:** 2020-08

**Authors:** Jae-Joon LEE, Chang-Hyun KANG

**Affiliations:** 1. Department of Health and Welfare, Graduate School, Dankook University, Cheonan, Korea; 2. Department of Public Administration, Dankook University, Cheonan, Korea

**Keywords:** Multiculturalism, Married migrant women, Childbirth intention, Number of childbirths

## Abstract

**Background:**

We aimed to identify factors related to childbirth intention in multicultural marriage migrant women in Korea. The study was based on the raw data of a National Survey on Multicultural Families 2015 in Korea, covering 7 countries and 31,047 participants.

**Methods:**

Data were analyzed with multiple regression analysis using SPSS and WIN 21.0 programs.

**Results:**

First, the consistent outcome in the seven countries was Residence period in Korea. The shorter the period of residence in Korea and the lower the age, the higher childbirth intentions. Second, the major factors according to country were Economic Activity, Satisfaction of Marital Relationship, Life Satisfaction, and Education. The most influential factors in each country were economic activity in Taiwan and Hong Kong, satisfaction with marital relationships in Korean-China, life satisfaction in Cambodia, and education in Mongolia and the Philippines. The higher the participation in economic activities, satisfaction with marital relationship, life satisfaction, and education level, the greater the number of childbirth intentions. Third, a contradictory result was found in State of health. In Vietnam, better health predicted greater numbers of childbirth intentions, while in China, Korean-Chinese, Mongolia, Cambodia, worse health predicated greater numbers of childbirth intentions.

**Conclusion:**

The findings suggest a need for a comprehensive multicultural policy and support services for multicultural marriage migrant women that considers characteristics such as country, cultural differences, and nationality in order to contribute to family formation and settlement of Korean society.

## Introduction

Modern industrial society in Korea has led to a decline in labor power and slower economic growth due to decreased fertility rates, an increase in the elderly population, migration between urban and rural areas, and changes in values, among other factors. To this end, in an effort to secure the economic population and resolve the socio-demographic structure, the Korean government promoted marriage with foreigners. Within these international marriages, domestic multicultural marriages accounted for 7.4% (22,462 cases) of all marriages in 2015, with 76.7% of married migrant women originating 27.9% from China, followed by 23.1% from Vietnam, and 4.7% from the Philippines (1). According to the statistics of multicultural populations published in 2015, although married immigrant women migrate from countries with high fertility rates and place importance on their children’s needs, the number of multicultural newborns was 19,729 in 2015 and has been steadily decreasing from 22,014 in 2012. Furthermore, the average number of multicultural children is 1.0, which is much lower than the average number of children born to Korean women at 1.75 (2).

Due to Korea experiencing low fertility, it is predicted that the low fertility rate of married immigrant women will have a substantial impact on demographic changes in Korea. In particular, to alleviate the low fertility rates of married immigrant women there has been a need to study not only factors affecting pregnancy and childbirth of married immigrant women in different cultural backgrounds according to each country, but also the intent to give birth to additional children and the number of these children (3, 4).

Additionally, changes in life related to sociodemographic, biological, psychological, economic, and socio-cultural(such as language, religion, and interpersonal relationships) factors accompanying the migration process and early settlement affect family formation, settlement, and child-birth of married immigrant women (5). Consequently, it is necessary to conduct research investigating the factors affecting the childbirth intention of married migrant women as a solution for the socio-demographic structure of Korea.

Recent quantitative and qualitative studies on pregnancy and childbirth for married immigrant women in Korea have indicated interest in the childbirth and number of children for married migrant women (1,3,4,6,7). However, these studies are limited in identifying specific factors relating to maternity and childbirth intentions for Korea’s married immigrant women originating from different countries for a particular area in Korea.

Therefore, as the purpose of this study is to identify the specific factors affecting the childbirth intentions of married migrant women from different originating countries in Korea, it is to provide a basis of data for preparing policy and support services for influencing childbirth and parenting activity of married migrant women to adapt to Korean society and to form a family.

## Materials and Methods

### Study Subject and data collection

This study used raw data from the National Survey on Multicultural Families 2015 in Korea performed by the Ministry of Gender Equality and Family and the National Statistical Office every three years to collect basic data required to establish policies for support of multicultural family members in accordance with the Multicultural Family Support Act. The survey was conducted from Jul–Aug in 2015.

The initial sample extracted 27,120 households and considered the local size and nationality of the survey population. Among them, 17,109 were married immigrants and naturalized persons. Married migrant women were 3,044 married immigrants and naturalized women who had the status of marriage immigrant status (F6) and Korean nationals’ spousal status (F-2-1). The study included 2,855 married migrant women from the top 7 countries (China, Korean-Chinese, Taiwan & Hong Kong, Mongolia, Vietnam, Philippines, Cambodia). Among them, those who responded insufficiently were excluded resulting in 2,062 married immigrant women aged between 19 and 39 who responded whether they planned to have children.

### Study Variable

In order to investigate the factors affecting the childbirth intentions of multicultural-marriage migrant women, 10 independent variables were identified with the number of children of childbirth intention as the dependent variable.

### Independent Variable

The independent variables consisted of the Age, Education, Residence period in Korea measured by demographic factors, Economic activity, Monthly income using socio-economic factors, Life satisfaction, Satisfaction with marital relationship, Participation in social activity, Difficulty living in Korea, and State of health of the multicultural marriage migrant women.

### Socio-Demographic Factors

Age was a continuous variable of 19–39 yr and Education was analyzed using the original data items classified as elementary school, middle school, high school, under 4 yr of college, over 4 yr of university, and higher than graduate school. The residence period in Korea was analyzed by calculating the residence period from the first entry year to the 2015 survey date.

### Socio-Economic Factors

In Economic activity, those who responded to 1–9 of nine categories of work related to economic activities were classified as ‘Yes’ for economic activity, non-responded were ‘No’ indicating they had no economic activity. Monthly income is the average monthly household income, and was analyzed by the original data items classified as 8 steps from less than 1 million won to over 7 million won, in units of 1 million won.

### Life Satisfaction

Life satisfaction was measured by the question: “How satisfied are you with your present life when you consider life as a whole?” The 5 point Likert scale of being very satisfied (1 point), slightly satisfied (2 points), moderately satisfied (3 points), not very satisfied (4 points), and not at all (5 points) was converted to reciprocal and it was analyzed that higher the score, the more satisfaction with life.

### Satisfaction with Marital Relationship

Satisfaction with marital relationship was measured with the question: “How satisfied are you with your spouse?” The 5 point Likert scale of being very satisfied (1 point), slightly satisfied (2 points), moderately satisfied (3 points), not very satisfied (4 points), and not at all (5 points) was converted to reciprocal and it was analyzed that the higher the score, the more satisfied with the relationship with your spouse.

### Participation in Social Activities

Participation in social activities was divided into five groups of participation experiences (Parents meeting; Friends group in your home country; Community gathering; Religious activity group; Private organization activity meeting). It was analyzed that ‘No’ participation experiences was 0 points and the sum of the items answered ‘yes’ was converted to 1 point.

### Difficulty Living in Korea

Difficulty living in Korea was classified into nine difficulties (Loneliness, Family conflict, Child care and education, Difficulty of using institutions such as banks and municipal governments, Economic difficulty, Language problems, Cultural differences such as lifestyle, customs, and food, Prejudice and discriminations, and Other questions). It was analyzed that ‘No’ difficulties was 0 points and the sum of the items answered ‘yes’ was converted to 1 point.

### State of Health

State of health was measured with the question: “What is your overall health condition?” The 5 point Likert scale of being very good (1 point), good (2 points), moderate (3 points), not well (4 points), and not very good (5 points) was converted to reciprocal and it was analyzed that the higher the score, the better overall health.

### Dependent Variable

As a dependent variable, the number of children in terms of childbirth intention is the number of children married immigrant women aged 19–39, as fertile women, included in response to the question: “How many do you plan to have (more)?” It was analyzed that plan to have “0” children was imputed as “No,” “Yes” was entered as a continuous variable for up to 5 persons, including women who were already pregnant.

### Study Analysis

To investigate the factors affecting the childbirth intentions of multicultural-marriage migrant women, multiple regression analysis was performed with age, education, residence period in Korea, economy activity, monthly income, life satisfaction, satisfaction with marital relationship, participation in social activities, difficulty living in Korea, and state of health as independent variables and number of children of childbirth intention as the dependent variable.

Because of verifying the Variance of Inflation Factor for all the variables of each country, no issue of multicollinearity of more than 10 was found (Table 1). In order to examine the outliers of this study, the cases with standardized residuals of more than 3.00 was China with 2 cases, Vietnam with 5, Mongolia with 2, Philippines with 6, and Cambodia with 2 and standardized residuals were 3.11–5.23.

**Table 1:** Factors related to childbirth intention of multicultural marriage migrant women in Korea

***Independent variable***	***China***			***Korean-Chinese***			***Taiwan & Hong Kong***		

	ß	t	VIF	ß	t	VIF	ß	t	VIF
Age	.200	15.300^***^	1.157	−.229	−9.707^***^	1.350	−.105	−3.804^***^	1.147
Education	.105	8.107^***^	1.136	.007	.275	1.422	.050	1.792	1.163
Residence period in Korea	−.212	−16.894^***^	1.069	−.076	−3.286^**^	1.309	−.164	−5.801^***^	−.200
Economy activity	.151	11.167^***^	1.234	.207	5.865^***^	3.010	.211	7.447^***^	1.205
Monthly income	−.028	−2.042^*^	1.270	−.033	−1.041	2.446	−.124	−4.488^***^	1.142
Life satisfaction	.135	8.890^***^	1.560	.155	6.303^***^	1.470	.089	2.350^*^	2.156
Satisfaction with marital relationship	.071	4.976^***^	1.395	.345	12.890^***^	1.729	−.018	−.507	1.989
Participation in social activities	−.152	−12.285^***^	1.043	−.130	−5.907^***^	1.171	.090	3.384^**^	1.054
Difficulty living in Korea	.033	2.623^**^	1.072	−.084	−3.853^***^	1.155	−.044	−1.559	1.211
State of health	−.033	−2.428^*^	1.220	−.478	19.424^***^	1.464	.003	.096	1.392
F		114.833^***^			90.813^***^			14.962^***^	
R^2^ / Adjusted R^2^		.169/.168			.375/.371			.100/.093	

Independent variable	Mongolia			Vietnam			Philippines			Cambodia		
	ß	t	VIF	ß	t	VIF	ß	t	VIF	ß	t	VIF
Age	−.058	−6.807^***^	1.190	−.151	−8.462^***^	1.429	−.033	−1.562	1.602	−.042	−1.593	1.816
Education	.117	14.778^***^	1.030	.110	7.012^***^	1.107	.182	10.002^***^	1.155	.128	6.199^***^	1.106
Residence period in Korea	−.265	−29.776^***^	1.292	−.166	−9.042^***^	1.503	−.293	−13.765^***^	1.583	−.313	−11.311^***^	2.000
Economy activity	.046	5.481^***^	1.162	−.022	−1.330	1.189	.018	.986	1.181	.121	5.631^***^	1.198
Monthly income	.071	8.762^***^	1.075	.023	1.438	1.173	.054	3.016^**^	1.124	.060	2.781^**^	1.236
Life satisfaction	.056	5.429^***^	1.726	.042	2.122^*^	1.730	.060	2.891^**^	1.518	.169	6.762^***^	1.635
Satisfaction with marital relationship	.016	1.600	1.671	.046	2.331^*^	1.711	.072	3.545^***^	1.455	.069	3.019^**^	1.362
Participation in social activities	.011	1.345	1.012	−.025	−1.684	1.019	−.126	−7.384^***^	1.027	−.085	−4.140^***^	1.110
Difficulty living in Korea	−.024	−3.058^***^	1.038	−.022	−1.468	1.052	−.008	−.471	1.037	.142	6.905^***^	1.100
State of health	−.032	−3.734^***^	1.178	.116	7.283^***^	1.140	.016	.886	1.159	−.147	−6.527^***^	1.322
F		200.382^***^			64.212^***^			64.657^***^			55.084^***^	
R^2^/Adjusted R^2^		.122/.122			.143/.141			.184/.182			.211/.207	

* *P*<.05,

** *P* <.01,

*** *P* <.001

VIF**:** Variance of Inflation Factor

Although these cases were classified as outliers, in this study, these cases were reflected and analyzed without excluding them due to the fact that multicultural marriage immigrant women have strong nationality and individual characteristics and the Korea National Statistical Office (KNOS), an investigative agency of the National Survey on Multicultural Families in 2015, has supplemented these limitations through adjustment of bias by using call-back, weighting, weight correction.

## Results

Table 1 shows the results of verifying the factors affecting the childbirth intentions of multicultural marriage migrant women.

By country, first, China explained 16.9% of the intention to give childbirth and the regression model was statistically significant. The most influential factors for the childbirth intention were residence period Korea (*ß* =−.212) and age (*ß* =−.200).

Second, Korean-Chinese explained 37.5% of the intention to give childbirth and the regression model was statistically significant. The most influential factors for the childbirth intention were state of health (*ß*=−.478) and satisfaction with marital relationship (*ß*=.345).Third, Taiwan & Hong Kong explained 10% of the intention to give childbirth, and the regression model was statistically significant. The most influential factors for the childbirth intention were economy activity (*ß*=.211) and residence period in Korea (*ß=−.*164).

Fourth, Mongolia explained 12.2% of the intention to give childbirth, and the regression model was statistically significant. The most influential factors for the childbirth intention were residence period in Korea (*ß=−.*265) and education (*ß=.*117).

Fifth, Vietnam explained 14.3% of the intention to give childbirth, and the regression model was statistically significant. The most influential factors for the childbirth intention were residence period in Korea (*ß=−.*166) and age (*ß=.−*151).

Sixth, Philippines explained 18.4% of the intention to give childbirth, and the regression model was statistically significant. The most influential factors for the childbirth intention were residence period in Korea (*ß=−.*293) and education (*ß=.*182).

Seventh, Cambodia explained 21.1% of the intention to give childbirth, and the regression model was statistically significant. The most influential factors for the childbirth intention were residence period in Korea (*ß=−.*313) and life satisfaction (*ß=.*169).

## Discussion

Within factors related to childbirth intention of multicultural-marriage migrant women originating from seven countries in Korea, first, common factors of Residence period in Korea and Age were consistent outcomes across the seven countries. In other words, the shorter the period of residence in Korea and the lower the age, the greater the number of childbirth intentions. This may be explained by women who migrated for marriage from a country where women have a high fertility rate and high need for children. This is consistent with previous finding (8) focusing on migrants and family bonding and interactions, suggesting that childbirth increases shortly after migration when forming a family, and findings (9) indicating that birth rates increase when the age at the time of marriage is younger.

Second, in terms of major factors and national aspects related to childbirth intention, the major factors are Economy activity, Satisfaction with marital relationship, Life satisfaction, and Education. By country, the most influential factors are economic activity in Taiwan & Hong Kong, satisfaction with marital relationship in Korean-Chinese, life satisfaction in Cambodia, and education in Mongolia and the Philippines. The findings indicate that the more economic activity participation, marital satisfaction, life satisfaction, and higher educational level, the greater the number of childbirth intentions. From these results, whilst the factor of economic activities corroborates with some previous studies (3, 10), it is contrary to others (11, 12) finding that female employment influences avoiding or delaying childbirth. Satisfaction with marital relationships relates to an extant study (6) indicating that the relationship quality with a husband affects childbirth. Life satisfaction is consistent with studies (5,13) in which the diverse stresses of life that can be experienced during the migration process such as language, culture, and social environment appear to affect lower fertility rate. Whilst education is consistent with some previous findings (14), it is inconsistent with another study (4) that it is not related to childbirth.

Examining each factor by country, in Taiwan and Hong Kong, as the economic activity became more participative, the intention to give birth increased. Traditionally, when males are the primary caregiver, they focus on economic activities while females are secondary caregivers who are devoted to childbirth and parenting activities. In developing countries where the division of gender roles in the family is well established, the fertility rate is said to increase (10). However, as Taiwan and Hong Kong are women’s rights-enhancing societies, women’s economic activities continue even after marriage, dual-income couples are common, and domestic labor is often shared within marriages (15). In this sense, it is believed that the economic activities of Taiwan and Hong Kong women have a significant impact on the planning of children.

In Korean-Chinese, the greater the satisfaction with marital relationships, the greater the intentions were to give birth. Women in Korean-Chinese have higher divorce rates than women in other countries when marrying a Korean man, many children are already born in China, and the remarriage rate is high. For this reason, as Korean-Chinese women tend to have little or no births after migration to Korea (16), it is suggested that a high level of marital satisfaction increases the plan to give birth.

In Cambodia, the higher the life satisfaction, the greater the intention of giving birth. It is consistent with studies (5, 13) finding that married migrant women have generally low birth rates due to the various stresses and difficulties in the lives they experience such as language, culture, and social environment during the migration process that deteriorates their life satisfaction. This was recognized as an important factor in the birth intentions of Cambodian women who married Korean men.

In Mongolia and the Philippines, the higher the education level, the higher the intention of giving birth. As Mongolia is a culture which has a socialist system and men have lower educational levels than women and work outside the home, whereas women are more educated than men and play a responsible role in the home, and Mongolian women who migrated for marriage migrated for better lives than the economic hardship in their home countries, they were highly motivated by their faithfulness to childbirth. In this fact, it led to a higher intention to give birth (17). In the Philippines, as migrant women with a high level of education are more active in childbirth intention in Korea, a low birth rate country (16), it is presumed that they accepted positively about the necessity of children and childbirth.

Third, as a contrasting factor, in the case of states of health, in Vietnam the better the health, the greater the number of childbirth intentions (18), while in China, Korean-Chinese, Mongolia and Cambodia, the worse the health, the greater the number of childbirth intentions. These countries are socialist countries with high gender equality and women status.

To begin with, in Vietnam where liberalization and capitalism are highly influenced, due to negative impacts on mental and physical health such as cultural adaption, family conflict, and child rearing this may decrease the quality of life and avoid birth (19).

Next, in China and Cambodia, the higher the level of education, the better the health and status of the women, and they may seek the qualitative aspects of their children, while the lower the level of education, the worse the health and status of the women and they may seek the number of children, the quantitative aspects (20, 21). Thus, these latter women who migrated for marriage with Korean men may exhibit a tendency to concentrate on giving birth and nurturing. However, in Mongolia, although women have high educational backgrounds, they may have migrated for better lives due to economic difficulties in their home county; in this respect, their daily mental and physical health conditions are degraded. Despite these circumstances, these women seem to have a high expectation of childbirth by positively accepting childbirth as a responsible role for their family in Korea, a low birth rate country (17).

Finally, it is notable that Korean-Chinese women who migrated to Korea for marriage have higher divorce and remarriage rates, avoid multiple childbirths and parenting, and their overall mental health level is poor due to high psychological anxiety and stress in everyday life such as excessive competition among Korean and Korean-Chinese women. Despite this, the calculation of cost benefit from interpersonal relationships, being a Korean through migration, and the expectation for the rise of the social and economic status the family as well as oneself can be assumed to increase childbirth intentions (16, 22).

## Conclusion

The intention of giving birth within Korea’s married immigrant women is influenced by various factors such as adaption to Korean society, burden on childbirth and care, economic situation, physical, psychological and mental health status, family relationships, cultural differences, and nationality among others. Accordingly, it is necessary to identify consistently these countries through various examples and follow-up studies. Therefore, these results will provide data based on the need for a comprehensive multicultural policy and support services for multicultural marriage migrant women contributing towards the settlement and family formation of Korean society. Eventually, the programs for married migrant women is needed to be customized based on scientific analysis because of differences by home countries and open immigration policy could be a path way to address low fertility rate in Korea.

## Ethical considerations

Ethical issues (Including plagiarism, informed consent, misconduct, data fabrication and/or falsification, double publication and/or submission, redundancy, etc.) have been completely observed by the authors.

## References

[1] Lee JY Kim YS (2016). 2015 Multicultural population dynamics statistics. Statistics Korea Daejeon

[2] Lee SS Park JS Lee SY (2015). The 2015 national survey on fertility and family health and welfare. Korea institute for Health and Social affairs Korea.

[3] Kim HG (2011). Cross-border married women’s fertility attitudes and behavior in Daegu Area. Inst Korean Cult, 49: 567–95.

[4] Yun SH Kim YH (2016). A study on childbearing of women of international marriage-based in North Gyeongbok province. Contemporary Society and Multiculture, 6(2): 94–123.

[5] Miewski N (2007). First child of immigrant workers and their descendants in West Germany. Demographic Res, 17(29): 859–96.

[6] Lim HS (2011). The experience of transition in pregnancy and childbirth and among the married immigrant women in Korea. Korean J Women Health Nurs, 17(3): 243–55. 10.4069/kjwhn.2011.17.3.243 37697552

[7] Cha SE Kim DS (2008). The effect of social roles on depression of foreign wives in Korea: Focused on the difference among Japanese, Chinese, Vietnamese wives. Korean J Popul Stud, 31(3): 131–57.

[8] Miewski N (2010). Immigrant fertility in West Germany: Is there a socialization effect in transitions to second and their births? Eur J Population, 26(3): 297–323.

[9] Chang KS (2013). Study on pregnancy, childbirth and child rearing of married immigrant women. J Multi-Cultural Contents Studies, 14: 341–65.

[10] Lee SY (2008). An analysis of the effect on childbirth will of married women. Korean Family Resource Management Association, 12(2): 15–30.

[11] Bae GI Kim GS (2012). A study on the influence of family values and birth policy on the wanted the fertility. Korean J Soc Welf Stud, 43(3): 239–66.

[12] Lee SS Jeon YJ Shin HY (2011). Analysis on impact of foreigners’ inflow in society undergoing low fertility and population age. Korea institute for Health and Social affairs Korea.

[13] Kulu H (2005). Migration and fertility: Competing hypotheses re-examined. Eur J Population, 21(1): 51–87.

[14] Min HJ Kim EJ (2011). The timing of births among Korean women: Parity difference in childbirths. Korean J Sociol, 45(4): 198–222.

[15] Lee IT (2001). Real china in China syndrome. Haenam Press Seoul.

[16] Kim SK Kim YK Choe AJ (2010). A national survey on multicultural families 2009. The Ministry of Justice & Ministry of Gender Equality Korea.

[17] Yun SJ The multicultural’ experiences of Mongolian migrant brides in South Korea [PhD, thesis]. School of Sociology, Yonsei University, Seoul; 2015.

[18] Kim YJ (2010). Childbirth and childcare policies for marriage migrant women and their characteristics. Korea J Popul Stud, 33(1): 51–73.

[19] Ryu JK (2017). Parity specific approach to the birth plan of foreign wives in Korea: Focusing on the effects of status mobility and proportion of foreign population. Health and Social Welfare Review, 37(1): 106–139.

[20] Li H Zhao MS (2007). Viewing the living condition of Chinese women from the change of fertility concept. J Texas College, 23(1): 49–53.

[21] Yoon CK Jang HN Moon SY (2016). The comparative analysis on QOL related to health of immigrant brides in urban and rural area. J Social Science, 14(2): 343–67.

[22] Gim MO Leisure desire and leisure behavior of married Korean-Chinese immigrant women [PhD thesis]. School of Leisure Tourism Development, Kyonggi University, Seoul ; 2010.

